# Do selective radiation dose escalation and tumour hypoxia status impact the loco-regional tumour control after radio-chemotherapy of head & neck tumours? The ESCALOX protocol

**DOI:** 10.1186/s13014-017-0776-1

**Published:** 2017-03-01

**Authors:** Steffi U. Pigorsch, Jan J. Wilkens, Severin Kampfer, Victoria Kehl, Alexander Hapfelmeier, Christian Schläger, Henning Bier, Markus Schwaiger, Stephanie E. Combs

**Affiliations:** 1 Department of Radiaton Oncology, Technical University of Munich, Klinikum rechts der Isar, Ismaninger Strasse 22, D-81675 Munich, Germany; 2 Münchner Studienzentrum (MSZ Coordination Centre for Clinical Trials), Technical University of Munich, Ismaninger Strasse 22, D-81675 Munich, Germany; 3 Institute of Medical Statistics and Epidemiology (IMSE), Technical University of Munich, Klinikum rechts der Isar, Ismaninger Strasse 22, D-81675 Munich, Germany; 4 Department of Radiation Sciences (DRS), Institut für Innovative Radiotherapie (iRT), Helmholtz Zentrum München, Ingolstaedter Landstrasse 1, D-85764 Oberschleissheim, Germany; 5 Department of Ear, Neck and Throat (ENT), Technical University of Munich, Klinikum rechts der Isar, Ismaninger Strasse 22, D-81675 Munich, Germany; 6 Department of Nuclear Medicine, Technical University of Munich, Klinikum rechts der Isar, Ismaninger Strasse 22, D-81675 Munich, Germany

**Keywords:** Dose escalation, Randomized prospective trial, Head and neck cancer, Chemo-irradiation

## Abstract

**Background:**

Standard of care primary treatment of carcinoma of locally advanced squamous cell head and neck cancer (LAHNSCC) consists of platinum-based concomitant chemo-irradiation. Despite progress in the treatment of LAHNSCC using modern radiotherapy techniques the outcome remains still poor. Using IMRT with SIB the escalation of total dose to the GTV is possible with the aim to improve clinical outcome. This study tests the hypothesis if radiation dose escalation to the GTV improves 2-year-LRC and -OS after concomitant chemo-irradiation.

**Methods:**

The ESCALOX trial is a prospective randomized phase III study using cisplatin chemo-irradiation and the SIB-IMRT concept in patients with LAHNSCC of the oral cavity, oropharynx or hypopharynx to escalate the total dose to the GTV up to 80.5 Gy. Chemotherapy is planned either in the 1^st^ and 5^th^ week (cisplatin 20 mg/m^2^/d d 1–5 and d 29–33) or weekly (cisplatin 40 mg/m^2^/d) during RT. RT is delivered as SIB with total doses of 80.5 Gy/70.0 Gy/56.0 Gy with 2.3 Gy/2.0 Gy and 1.6 Gy in the experimental arm and in the control arm with 70.0 Gy/56.0 Gy with 2.0 Gy and 1.6 Gy. A pre-study with dose escalation up to 77.0 Gy/70.0 Gy/56.0 Gy with 2.2 Gy/2.0 Gy and 1.6 Gy is demanded by the German federal office of radiation protection (BfS). In the translational part of the trial 100 of the randomised patients will be investigated by 18-F-FMiso-PET-CT for the presence and behaviour of tumor hypoxia twice in the week before treatment start.

**Discussion:**

The primary endpoint of the pre-study is acute radiation induced toxicity. Primary endpoint of the main trial is 2-year-LRC. By using the dose escalation up to 80.5 Gy to the GTV of the primary tumor and lymph nodes > 2 cm a LRC benefit of 15% at 2 years should be expected. The ESCALOX trial is supported by Deutsche Forschungsgemeinschaft (DFG); Grant No.: MO-363/4-1.

**Trial registration:**

ClinicalTrials.gov Identifier: NCT 01212354, EudraCT-No.: 2010-021139-15

## Background

Head and neck cancers are the seventh most frequent cancer in Germany (estimated incidence of 50/100.000 per year, Deutsche Krebsgesellschaft). The German Robert-Koch-Institut presented recently data on absolute disease numbers of all tumor entities. In 2013 for head and neck cancer there were 4.500 women and 13.000 men newly diagnosed in Germany [1]. For 2020 the new diagnosis expectations are higher: women 5.500 and men 14.300. Worldwide there are more than 550.000 new cases every year [2].

Head and neck cancers are representative for solid tumours in which new biological-imaging-based treatment strategies can be tested comparatively easily. This is true especially with regard to the impact of tumour volume, which correlates with cell number per tumour and tumor hypoxia, on response after non-surgical treatment.

Concomitant chemo-irradiation is superior to radiation alone in inoperable LAHNSCC. An absolute survival benefit of 8% at 5 years favouring chemo-irradiation was shown in the meta-analysis of the MACH-NC-group (2000) [3]. The up-date of the MACH- NC-group in 2009 included studies between 1994 and 2000. Again there was an absolute benefit of 6.5% for chemo-irradiation (independent of the setting – postoperative or primary chemo-irradiation) concerning the HR of death in comparison to local treatment alone [4]. In 2011 the MACH-NC group investigated the effect of chemo-irradiation in comparison to radiotherapy and revealed a 5-year absolute benefit (OS) due to concomitant chemotherapy for oral cavity: 8.9%, oropharynx: 8.1% and 4% for hypopharynx [5].

Besides tumor volume correlating with cell number per tumor, hypoxia is a further important biological parameter for tumor progression. Hypoxia increases radio-resistance and is a predictive factor for local failure. Hypoxia occurs in about 80% of head and neck tumors (primaries and/or lymph nodes). Based on experimental and clinical data, hypoxia is considered as a useful parameter for pre-therapeutic stratification in future randomized chemo-irradiation trials. More importantly, hypoxic sub-volumes of tumors are also evolving as target volumes for radiotherapy (“dose painting”) in some studies examining the significance of selective irradiation dose escalation. To investigate the shift of the hypoxic sub-volumes [18 F]-FMISO PET will be performed twice within 1 week before start of radiation treatment.

### Hypothesis of the trial

Over the last decade IMRT became a new standard in radiotherapy. Different trials used the advantage of IMRT concerning target volume coverage and protection of organs at risk. Analyses of acute toxicity of IMRT revealed advantage for lower toxicity compared to 3D-conformal radiotherapy with comparable treatment results.

The 2-year local control rate varies between 77–98% and the loco-regional control at 2 years between 86%–100% for non-selected tumor stages [6–8]. Two studies reported 2-year overall survival between 90–94% [9, 10] for patients with locally advanced disease.

The ESCALOX trial investigates whether dose-escalated SIB-IMRT to the primary tumor and involved cervical lymph nodes (≥2 cm) with concurrent cisplatin chemotherapy improves loco-regional control within 2 years by 15% in comparison to conventional SIB-IMRT with concurrent cisplatin.

The experimental arm of the ESCALOX-trial has not yet been tested in a multi-institutional setting. Taking into account all the mentioned results on acute and late toxicity it seems possible to apply dose escalated radiotherapy with optimization of the IMRT-plan concerning the organs at risk.

## Methods/design

### Trial organization/coordination

ESCALOX is a prospective randomized multicentre national phase III trial. The performance of the study is supported by Deutsche Forschungsgemeinschaft (DFG; MO- 363/4-1). ESCALOX is an investigator initiated trial (IIT) and is coordinated by the Department of RadioOncology and Radiotherapy of the Technical University of Munich. The Münchner Studienzentrum (MSZ) at the Technical University of Munich (Munich trial coordinating centre) is responsible for overall trial management, trial registration, database management, quality assurance including monitoring and reporting. The radio-oncological quality assurance (delineation and treatment planning) is centrally performed by the departments of radiation oncology of the two university hospitals in Munich (Technical University of Munich and Ludwigs-Maximilians-University Munich).

### Ethics, informed consent and safety

The final protocol was approved by the ethics committee of the Medical Faculty of the Technical University of Munich (2847/10 Af) and the German Federal Institute for Drugs and Medical Devices (Bundesinstitut für Arzneimittel und Medizinprodukte - BfArM; BfArM-registration number 4036421) and the German Federal Office of Radiation Protection (Bundesamt für Strahlenschutz – BfS) (BfS-registration number Z5-22463/2-2011-011).

Written informed consent is obtained from each patient in oral and written form before inclusion in the trial. Nature, scope and possible consequences of which will been explained by the responsible physician.

An independent safety board of four experts was named (three of them are radio-oncologists, one statistician). After the end of the pre-study as recommended by the German Federal Office of Radiation Protection a safety report will be sent to the BfS. During this phase there will be a stop of 6 months of the trial until the BfS gives permission for further recruitment of patients.

### Study design

**Fig. 1:**
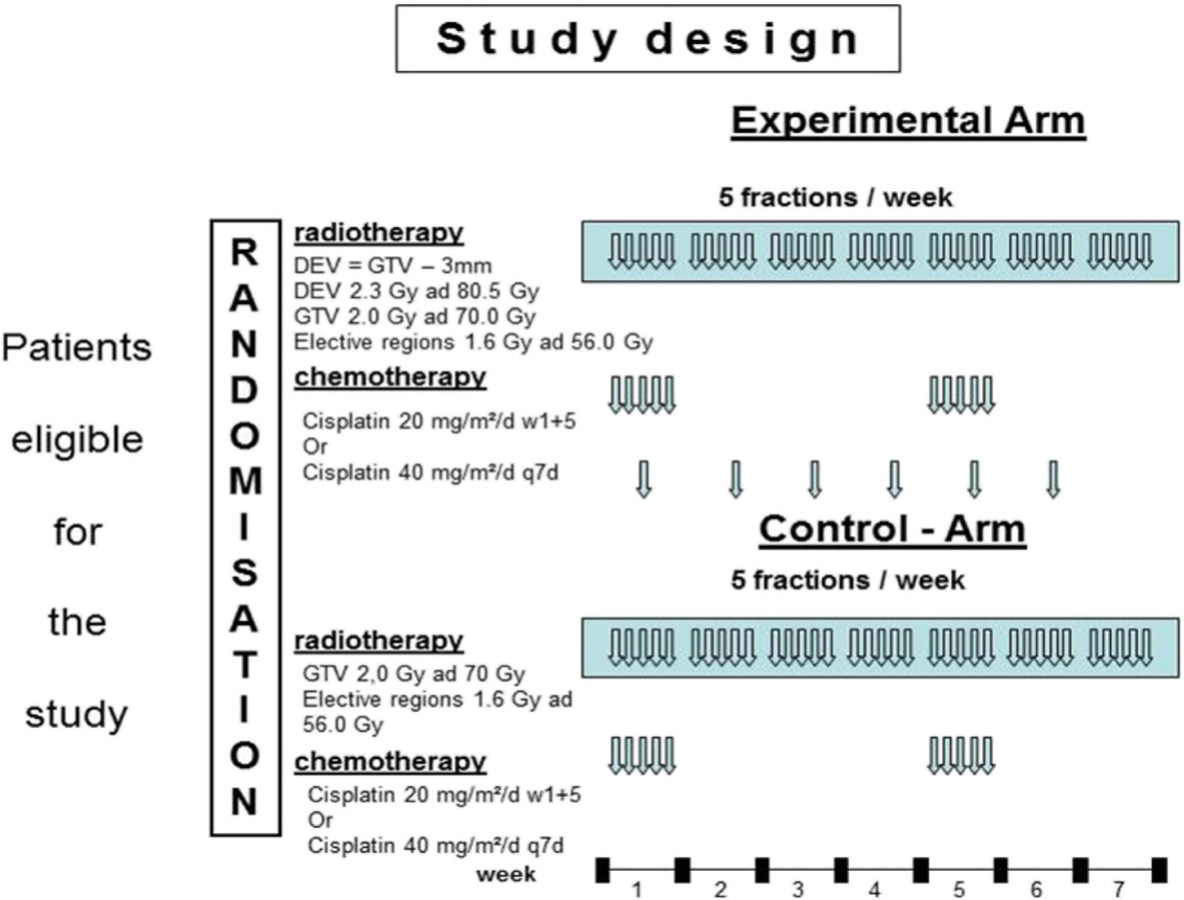
ESCALOX - Trial scheme

The ESCALOX trial is an investigator initiated (IIT), rater-blinded, multicentre national phase III trial combining dose escalated radiotherapy of macroscopic tumor in LAHNSCC with the standard cisplatin chemotherapy (Fig. 1). The German Federal Office of Radiation Protection (BfS) demanded the enforcement of a pre-study to check the safety of a dose-escalation trial concerning the acute toxicities in a first step up to 77.0 Gy for twenty patients.

IMRT or VMAT will be performed to apply the RT as simultaneous integrated boost. The ESCALOX trial enrols patients with LAHNSCC of the oral cavity, oropharynx or hypopharynx. Patients will be treated by the University Departments of Radiation Oncology and Radiation Therapy in Germany.

The primary endpoint of the pre-study as recommended by the German Federal Office of Radiation Protection (BfS) will be the assessment of acute toxicity. The primary endpoint of the main study is the local-regional control (LRC) at 2 years.

The secondary endpoints are disease-free survival (DFS), progression-free survival (PFS), overall survival (OS), acute and late radiation effects, adverse events, tumor hypoxia status investigated by 18-F-FMiso-PET.

### Patient selection

As defined by the German federal office of radiation protection a pre-study is performed in a step wise design. In the pre-study 20 patients will be included. The inclusion criteria are the same. For the main study a total of 250 subjects with LANHSCC are planned to recruit. For inclusion and exclusion criteria see Table 1.

**Table 1:** Inclusion and Exclusion criteria of the ESCALOX-trial; (*) A tumour is classified as non-resectable if the probability of a complete tumor resection is estimated at a very low level (non-R0). The decision to classify a tumor as non-resectable has to be confirmed by consent of ENT-specialist, oral surgeon, radio-oncologist, radiologist and pathologist. Detailed criteria of irresectability see Adelstein et al. [55]

Inclusion criteria	Exclusion criteria
▪ Signed written informed consent ▪ Age ≥ 18 ≤ 70 years ▪ Independent of gender ▪ Independent of race ▪ ECOG 0 – 2 ▪ Tumor of oral cavity, oropharynx or hypopharynx ▪ Histology: squamous cell carcinoma, HPV-negative ▪ Curative treatment intended ▪ Tumor is classified as irresectable [11]* ▪ Woman of child-bearing age: negative pregnancy test in serum ▪ Contraception in male and female patients and their partners if of childbearing potential, willingness to use effective contraceptive method for the study duration and 2 months post therapy ▪ Sufficient bone marrow reserves during 7 days before study inclusion; (leukocytes ≥ 4 × 10^9^/l, absolute no. of neutrophiles (ANC) ≥ 2 × 10^9^/; ▪ thrombocyte count ≥ 100 × 10^9^/l; hemoglobin ≥ 10 g/dl) ▪ adaequate liver function during 7 days before study inclusion (total bilirubine ≤ 2,5 × ULN (upper limit of normal), ASAT/ALAT ≤ 2,5 × ULN, ▪ alkaline phosphatase ≤ 2,5 × ULN of the institution’s normal value) ▪ adequate kidney function during 7 days before study inclusion; serum creatinine ≤ 130 μmol/l; creatinine clearance ≥ 70 ml/min ▪ all patients should have a dental examination before starting therapy and if necessary be treated, adaptation of a teeth protection bar ▪ a percutane feeding tube should be applied before start of treatment	▪ Infiltration of the mandible and/or larynx ▪ HPV-positive proven tumors ▪ impaired renal and/or liver function ▪ secondary malignancy, unknown primary cancer, nasopharynx cancer or ▪ salivary gland cancers ▪ Metastatic disease ▪ Another cancer within 5 years of study entry ▪ Serious concomitant disease or medical condition ▪ Pregnancy or lactation ▪ Women of child-bearing potential with unclear contraception (postmenopausal ▪ women must have been amenorrheal for at least 12 months to be considered of non-childbearing potential) ▪ previous treatment with chemotherapy, radiotherapy or surgery in head and neck (except an excisional biopsy or biopsy for histology and surgery for benign disease) ▪ concurrent treatment with other experimental drugs or participation in another clinical trial with any investigational drug within 30 days prior to study screening ▪ life expectancy of < 1 year ▪ contraindications to receive Cisplatin ▪ social situations that limit compliance with study requirements

### Pathology

From the specimen taken during pan-endoscopy the tumor histology and HPV16 status will be determined.

### Imaging

During screening the standard staging program to exclude lung and liver metastasis or secondary cancer is performed by CT Thorax and ultrasound of the liver.

CT or MRI of the head and neck is performed at the time of screening and then at every follow-up visit using the RECIST-criteria version 1.1.

## Safety

### Safety pre-study

The federal office of radiation protection (BfS) requires the successful completion of a pre-study before the main study is allowed to recruit patients.

The pre-study is planned to be carried out as follows:

A pre-study was initiated before starting treatment according to ESCALOX’s planned study arms (arm A: the experimental group receiving a dose escalation up to 80.5 Gy and arm B: the control group receiving a standard dose up to 70.0 Gy).

The pre-study is analogue to the experimental group A but the dose-escalation to the macroscopic tumor and involved lymph nodes ≥ 2 cm is restricted to 2.2 Gy up to a total dose of 77.0 Gy in this SIB-volume.

In the first step 6 patients will be treated up to 77.0 Gy. After the last patient finished the RT-course there was an interval of 6 weeks defined to assess acute toxicity. When the last patient has finished the 6 weeks of follow-up and no more than 1 of the 6 patients reached grade 4 radiation induced toxicity (CTCAE v. 4.0), further 14 patients will be allowed to enter the pre-study. For all of these 20 patients the 6 weeks follow-up is fixed to assess acute toxicity as mentioned above. If no more than 2 out of all 20 patients reached grade 4 toxicity (CTCAE v. 4.0) at this time (6 weeks after end of treatment) recruitment for the main part of the ESCALOX trial with randomization into arms A and B is allowed to begin.

### Safety main study

Randomization of patients into the main part of the ESCALOX trial is only possible after successful completion of the pre-study.

For the main part of the trial there are the same conditions determined by the German federal office of radiation protection (BfS) for a step wise recruitment of patients to the experimental arm as for the pre-study. Six patients per study arm will be allowed to be treated (arm A in the SIB80.5Gy-volume up to 80.5 Gy and in arm B in the SIB70Gy-volume up to 70 Gy). “Early” follow-up for these patients is defined with 6 weeks after the end of therapy. If the last patient has finished the 6 weeks of follow-up and no more than 1 of the 6 patients in arm A (dose escalation up to 80.5 Gy) reached grade 4 radiogenic toxicity (CTCAE v. 4.0), then further 14 patients per arm will be allowed to be treated. Patient “early” follow-up will continue for at least 6 months after radiation treatment has ended.

After the end of this 6 months period all toxicities have to be reported to the BfS. Only with a new permission of the BfS further patients can be recruited and treated in the ESCALOX trial.

### Adverse event (AE)

Radiotherapy-related toxicities will be assessed using the NCI Common Toxicity Criteria (CTCAE v.4.0).

### Serious Adverse Events (SAE)

In this trial the following are not to be classified as Serious Adverse Events:

- Admission to hospital required by the protocol
- Admission to hospital for management of the underlying disease
- Disease progression. However if, due to disease progression, a death occurs during the reporting period, it should be reported as SAE.
- Myelo-suppression, impaired renal function after cisplatin chemotherapy, acute side effects of radio-chemotherapy (such as fever, infection, bleeding and related hospitalizations, mucositis, radiodermatitis, dysphagia, odynophagia).

These events are expected events in the treatment of malignancies of the head and neck region. They should be reported as “Adverse Event “, but should not be reported as SAE.

## Quality assurance

### Dummy run/RT QA

Before the initiation of a trial centre each site has to perform a “dummy run” to demonstrate that the centre complies with the specific study requirements for delineation, IMRT planning, dose distribution and treatment delivery. For the dummy run a planning CT will be sent by the IMRT-planning QA centre to each participating institution and the results (structures, isodoses, DVH’s) are evaluated by the QA centre. When the dummy run is successfully completed, a site will be allowed to recruit patients for the study. The first 3 patients of a trial site will be checked for centrally QA.

## Study treatment

### Chemotherapy

The chemotherapy of the ESCALOX trial will contain a platinum based protocol. The application of Cisplatin 20 mg/m^2^/d in week 1 (d1-5) and 5 (d29-33) as well as Cisplatin given in a weekly schedule with 40 mg/m^2^/d q d7 (max. 6 cycles) is allowed. In the case of impairment of renal function due to cisplatin the switch to carboplatin with AUC 2 in a weekly application scheme is allowed.

### Radiation therapy

The use of linear accelerator or a tomotherapy machine with nominal photon energy ≥ 6 MV is allowed. Electron beams are prohibited. The recommended photon energy is 6 or/and 10 MV. Only IMRT with SIB-technique is allowed. All fields of the IMRT-plan will be treated on every treatment day. It is recommended to avoid beams through sensitive structures (OARs) i.e. the eyes. The radiotherapy course has to be completed with 35 fractions in 55 days (with holidays, weekends, planned interruptions: day off, machine maintenance).

### Re-treatment planning

In all cases a second and a third planning CT should be done after 15 and 25 fractions of radiotherapy. Re-Planning has to be done if changes of the GTV_PT+LN_ are clinically significant. If there is > 5% loss of body weight and/or > 1 cm difference in any set-up parameter during treatment a re-planning CT should be performed and the treatment plan has to be adapted. The use of image guidance is recommended.

### Planning priorities

- The planning target volumes (SIB 56, SIB 70 and (SIB 77) SIB 80.5) should be delineated in the way that there is a sufficient level of homogeneous tissue for adequate dose homogeneity within the PTVs.
- For the OAR constraints the biological equivalence dose for 2 Gy (EQD2) using the α/β – value of 3 is calculated.

The PTV prescription dose followed by the OAR-constraints (see Table 2) has the highest priority.

**Table 2:** ESCALOX - trial Dose constraints for Organs at Risk (OAR)

OAR	Necessity for planning	accepted dose [EQD2]
Spinal cord (PRV: Myelon + 5 mm)	Mandatory	D_max_ < 50 Gy_2_ or No more than 1 cm^3^ > 45 Gy_2_
Brachial plexus	Mandatory	60 Gy_2_
Brainstem	Mandatory	D_max_ < 54 Gy_2_ or no more than 1 cm^3^ > 54 Gy_2_
Optical nerve	Mandatory	Dmax < 54 Gy_2_
Mandible	Recommended	D_max_ < 70 Gy_2_ or no more than 1 cm^3^ > 70 Gy_2_
Glottis (outside PTV)	Recommended	2/3 < 50 Gy_2;_ D_mean_ < 45Gy_2_
Gl. parotis	Recommended	D_mean_ < 26 Gy_2;_ D_50_ < 30 Gy_2_ 20 cm^3^ of both < 20 Gy_2_

a) Dose prescription of SIB 80.5, SIB 70, SIB 56.

b) OAR (spinal cord, brainstem, Glottis, mandible; in this order).

c) Further aim of the planning: total dose to the salivary glands as low as possible.

### Target volumes and dose prescription

#### Target volume definitions and delineation for control and experimental group (main study)

***Gross tumor volume*** *(* ***GTV)*** **:** The description of the gross tumor volume is based on CT and optionally MRI scan, endoscopic and clinical examinations as well as additional imaging.

A lymph node should be classified as involved, if it has/there are

- diameter ≥ 1 cm (jugulo-digastric lymph nodes >1.1–1.5 cm) in the first echelon of the tumor
- diameter ≤ 1 cm with spherical shape in the first echelon of the tumor
- lymph nodes containing necrotic areas
- multiple (minimal 3) lymph nodes of the ipsilateral neck of the primary tumor.

All these lymph nodes should be delineated for GTV.

***Clinical target volume (CTV)*** is defined as the GTV plus regions classified to be at risk for microscopic spread. The extension of this volume is determined by the radio-oncologist.

**CTV 70** is defined as GTV of the primary tumor plus 1 cm and GTV of the involved lymph nodes plus 0.5 cm but for cranio-caudal direction also 1 cm (for control group and for experimental group).

**CTV 56** is defined as elective lymph node levels (for control group and for experimental group).

***Planning target volume (PTV)*** here deemed as SIB 56 and SIB 70 for experimental and control group.

**SIB 56** is defined as an additional set-up margin around CTV 56 in all directions to compensate for morphological changes (i.e. weight loss), set-up deviation and internal organ motion.

0.5 cm as safety margin is recommended for the upper cervical structures and 0.7 cm for the lower cervical structures. Exceptions are the direct neighborhood of critical organs as the spinal cord or other organs at risk.

A skin sparing of 0.3 cm is recommended unless the tumor invades near the skin or e.g. into the sternocleidomastoid muscle.

In addition, the set-up margin should be defined according to the results on set-up error for IMRT at each participating center.

The **SIB 70** will be derived from the CTV 70 (automatically contoured margins by the planning system) as described above. The SIB 70 has to be adapted by hand at each planning CT slice to organs at risk and anatomical boundaries.

#### Pre-Study: Special Definition of dose escalated planning target volume (SIB 77.0) for the pre-study group in step-wise recruitment

**Dose escalated volume (deemed as SIB 77.0):** The dose escalated volume is defined as the gross tumor volume (GTV) of the primary tumor (PT) and all involved lymph nodes (LN) with a minimal axial diameter > 2 cm. At critical structures (e.g. mucosal sites, vessels, skin) the GTV is reduced by 0.3 cm (inner margin). Treatment planning will be performed without set-up error for the SIB 77.0.

All lymph nodes detected as involved with a minimal axial diameter of 2 cm will be also included as SIB 77.0.

For definition of SIB 70 and SIB 56 see above (Planning target volume (PTV) here deemed as SIB 56 and SIB 70).

##### Special Definition of dose escalated planning target volume (SIB 80.5) for the experimental arm (group A)

**Dose escalated volume (deemed as SIB 80.5**)**:** The definition for the SIB 80.5 uses the same guideline as for SIB 77 in the pre-study. Planning will be performed without set-up error for SIB 80.5.

Description of delineation of SIB 70 and SIB 56 is given above.

The dose constraints for organs at risk (OAR) are shown in Table 2.

### Evaluation of therapy outcome

CT or MRI of the head and neck is performed at the time of screening and then at every follow-up visit (4 month interval during the first two follow-up years) using the RECIST-criteria version 1.1 to assess efficacy of chemo-irradiation.

## Statistics

### Sample size calculation

Sample size calculations are based on the Log-rank test for superiority of the experimental arm over the comparator arm in LRC-survival over two years. The two-year LRC-survival is expected to be about 60% in the comparator group and about 75% in the experimental group. When the sample size in each group is 125, the one sided Log-rank test will have power of about 80% to detect the assumed difference at the 0.05 level. Therefore, 250 patients need to be recruited in the trial, 125 in each arm.

Due to missing alternative therapy concepts in non-resectable LAHNSCC, the expected compliance rate will be over 95%. Missing values will be conservatively treated: In the control arm, missing values will be imputed by the longest observed loco-regional survival time. In the experimental arm, missing values will be set to the time of censoring.

Because of the pre-study requirements of the BfS the pre-study will include 20 patients. These 20 patients are on top of the calculated 250 patients for the whole trial.

### Statistical methods

#### Primary efficacy endpoint analysis for the main part of the trial

The primary efficacy endpoint of the trial, LRC-survival over two years, will be tested for superiority of the experimental arm over the comparator arm using the one-sided Log-rank test at the 0.05 significance level on the ITT set. LRC-survival is defined as time to local relapse or death calculated from start of radiotherapy.

The corresponding hypotheses are:

H0: Se(t) ≤ Sc(t) vs. Ha: Se(t) > Sc(t),

Se and Sc denote the two-year LRC-survival in the experimental arm and in the control arm respectively.

#### Secondary efficacy and safety endpoint analyses

Secondary efficacy and safety endpoints will be analyzed in an explorative manner on a 5% significance level. Kaplan-Meier estimators and life-table methods will be used for the analysis of time-to-event data and life-table data. Cumulative incidence estimators will be used in case of competing risk. The reproducibility of PET hypoxic regions will be evaluated as the proportion of overlapping voxels. Continuous endpoints will be analyzed with the *t*-test (Mann–Whitney-*U* test if not normal). Categorical endpoints will be analyzed with the Chi-squared test (Fisher’s exact test if the expected cell frequency is less than 5). Repeated measures will be analyzed by paired-samples *t*-test, Wilcoxon singed-rank test or mixed models.

#### Missing data

Missing values will be conservatively imputed for the assessment of the primary endpoint: In the control arm, missing values will be imputed by the longest observed loco-regional survival time. In the experimental arm, missing values will be set to the time of censoring. The primary endpoint will also be analyzed as a secondary endpoint without imputation. Imputation is not performed for the secondary endpoints.

### Interim analysis plan

On behalf of the BfS-requirements interim safety analysis as described above are necessary. For the safety interim analysis after the first 60 patients neither the primary nor the secondary endpoints will be assessed.

Primary endpoint will be assessed after two year follow-up of the last included patient.

## Discussion

The addition of concomitant chemotherapy to radiotherapy, irrespective of fractionation schedule, increases overall survival by 12 months [11]. In the 2011 meta-analysis of the MACH-NC-group the additional effect of chemotherapy concerning HR of death was calculated to be 0.88 [5]. 5-FU and cisplatin or cisplatin mono based concomitant chemo-irradiation gained the best survival benefit compared with other cytostatic drugs [4].

The chemotherapy treatment of the ESCALOX trial will use cisplatin weekly with 40 mg/m^2^/day or cisplatin in the 1^st^ and 5^th^ week of the radiotherapy given on 5 days with a cumulative dose of 200 mg/m^2^ in both schedules. At this time there is still controversial debate on the best cisplatin regimen weekly vs. three weekly [12] and on the number of cycles [13].

In spite of the significant clinical benefit from concomitant radio-chemotherapy, there is still the need for further improvement. Two and 5 year loco-regional control rates after combined therapy are given from 40% to 65%. These disappointing numbers indicate that most loco-regional recurrences are observed within the first 2 years after treatment and that overall survival considerably declines with further follow up [14]. The latter is also the result of a high likelihood of non-cancer deaths related to the smoking and drinking habits of many head and neck cancer patients [15]. The overall survival in the same trials at 2 and 5 years ranges between 50% to 65% and 35% to 45, respectively. The absolute gain of 5-y-OS is 6.5–8% due to concomitant chemotherapy [4, 5].

In clinical trials testing chemotherapy in combination with identical radiotherapy regimens in all arms of the study, loco-regional control does not correlate well with overall survival. In contrast, in randomized trials addressing radiation dose or fractionation schedules loco-regional control has been shown to be a reliable surrogate marker of overall survival [14]. Any improvements in overall survival by dose escalation of radiotherapy with constant overall treatment time has to be attributed to an increase in clonogenic tumor cell kill within regions receiving an escalated dose, and in this way caused by better loco-regional control.

Data on long term outcome of different fractionation schedules are now available. The long term results of the RTOG 90–03 trial presented the hyper-fractionation arm with twice daily 1.2 Gy up to 81.6 Gy having the best results concerning 5 y OS (HR = 0.81) and 5 y LRC (HR = 0.79) [16]. This trial compared 4 fractionation schedules without chemotherapy: hyper-fractionation (HF) with 1.2 Gy twice daily up to 81.6 Gy; normo-fractionation (NF) of 2 Gy up to 70 Gy; accelerated radiotherapy (AF) with split (1.6 Gy twice daily up to 38.4 Gy – two weeks break – 1.6 Gy bid up to 67.2 Gy) and the concomitant boost schedule (AF + ccb) with 1.5 Gy and 1.8 Gy up to 72 Gy. At 2 years HF and AF + ccb yielded best loco-regional control. In comparison to NF all patients treated by altered fractionation schedules developed more acute side effects [17]. The long-term follow-up data of RTOG 90–03 showed superiority of the HF scheme (81.6 Gy) concerning 5y-OS HR =0.81 and 5y-LRC HR = 0.79; *p* = .05). Acceleration appeared to increase the 5y toxicity [16].

Budach et al. [18, 19] compared in the German ARO 95–06 study two hyper-fractionated accelerated fractionation schedules with chemotherapy (5-FU and Mitomycin C) 70.6 Gy and without chemotherapy but a dose escalation up to 77.6 Gy concerning the loco-regional control. The 5- and 10-year results favored the concomitant chemo-irradiation arm with 49.9%/38.0% vs. 37.4%/26.0% for loco-regional control and also for overall survival with 28.6%/10% vs. 23.7%/9%. In the 10 year analysis there was the remark that the association between treatment arm and LRC was only found for oropharyngeal cancer.

Considering the results of the ARO 95–06 [18, 19] and the RTOG 90–03 [16, 17] it is possible to apply a total dose of 77.6 to 81.6 Gy hyper-fractionated (accelerated) to the head and neck region concerning the normal tissue tolerance. In the ARO 95–06 trial the dose escalation alone could not outweigh the effect of omitting concomitant chemotherapy. Both trials used 3D-conformal radiotherapy. By radiobiological modelling was shown that the combination of chemotherapy reaches an improvement of LRC which is not possible by radiotherapy dose-escalation alone [20, 21].

The acute toxicities of the ARO 95–06 trial were for the radiotherapy arm (77.6 Gy) concerning mucositis III°/IV° 75.7% and for the radio-chemotherapy arm (70.6 Gy) 65.7%. Grade III and IV acute radiodermatitis occurred in 46.3% (radiotherapy alone) vs. 29.6% (radio-chemotherapy) using photon and electron techniques. The amount of toxicity should be decreased by using IMRT in the presented protocol as reported in many publications on IMRT approach in head and neck cancer RT.

Fu et al. reported a grade III acute mucositis of 25% for 70 Gy (normo-fractionated - NF), 41% for 81.2 Gy (hyper-fractionated accelerated - HF) and 46% for 72 Gy (accelerated fractionated with concomitant boost – AF + ccb). A mucositis of grade IV developed in only 0–1% of all patients. The acute xerostomia grade II rate was comparable between all groups (64–72%). Over all patients of the RTOG trial 90–03 mainly a grade II acute radiodermatitis developed (49–55%). Seven percent of the normo-fractionated (NF) group (70 Gy) and 11% of the hyper-fractionated (HF) respectively the accelerated concomitant boost (AF + ccb) group suffered from acute radiodermatitis of grade III [17].

From literature it is known that late toxicities of the above mentioned therapy schemes are comparable. Over the last decade there were 3 randomized trials performed comparing IMRT with conventional radiotherapy [22–24]. The primary endpoint of all trials was the development and severity of xerostomia. In a randomized controlled setting the hypothesis of reducing xerostomia by IMRT was verified. Focusing clinical outcome (LRC and OS) all three trials revealed comparable results. But none of the trials were powered to detect differences in clinical outcome data.

Data from published series on simultaneous integrated boost technique for locally advanced head and neck cancer using single doses between 2.2 Gy – 2.4 Gy up to a total dose range from 60–70 Gy showed tolerable acute side effects. Guerrero Urbano et al. 2007 [25] and Schwartz et al. 2007 [26] used single doses of 2.4 Gy up to 60–67.2 Gy. Schwartz et al. reported on 49 patients of whom 92% completed therapy without interruption. The majority (59%) of the patients had concomitant chemotherapy. The only grade IV acute toxicity was related to dysphagia (acute mucositis I° 12%, II° 32%, III° 55%; acute dysphagia I° 40%, II° 14%, III° 24%; acute xerostomia I° 36%, II° 12%, III° 3% and acute radiodermatitis I° 3%, II° 30%, III° 59%; weight loss in 22% of patients). In one patient an osteoradionecrosis occurred. Guerrero Urbano et al. [25] reported on 87% of stenosis or strictures of the esophagus.

De Arruda et al. (2006) [27] reported on 72% grade III and IV acute mucositis and 20% grade III and IV acute radiodermatitis using 2.2 Gy per fraction up to 70 Gy combined with concomitant cisplatin.

The acute mucositis III° and IV° is comparable between the series by de Arruda et al. (2006) [27] and the ARO 95–06 trial by Budach et al. (2005) [18]. Most of the SIB-trials using total doses about 70 Gy reported on ~ 50% acute mucositis III°.

Studer et al. (2006) [9] used three different SIB schedules (2.0, 2.11 or 2.2 Gy per session) and concluded that a SIB single dose of 2.2 Gy is not recommended for tumors involving laryngeal structures (two IV° reactions (dysphagia, laryngeal fibrosis)).

Most concern by using SIB-concepts with dose escalation is the development of late mucosal ulceration. In some cases described these ulceration arose 3 to 10 months after the end of radiotherapy [28–31]. The ulcerations were often located in the primary tumor region. Interestingly, in some cases a spontaneous healing was seen. Some of the patients who developed mucosal ulceration had persistent alcohol and tobacco abuse after the end of therapy. Because of these findings the German Federal Institution of Radiation Protection recommended to perform a pre-study to investigate the safety of the planned dose escalation in two steps.

Distinct dose response curves for radiotherapy have been calculated from clinical studies on head and neck cancer [20]. According to this data, one would expect an improvement of loco-regional control of an average 10% (range 6.9–40%) by a dose escalation of 10%. However, the available data are consistent with the hypothesis that the slope of dose response in large tumors is considerably shallower. The latter is likely related to the relatively larger hypoxic areas in large tumors [32, 33].

Generally, in head and neck tumours hypoxia plays a major role. In clinical radiation oncology it could be proven that hypoxia is correlated with a worse prognosis in especially in head and neck cancer [34–36].

Hypoxia reduces radiation- and chemo-sensitivity to a considerable extent as shown also in many experimental investigations. Using PET imaging and appropriate markers, hypoxia is found in about 80% of patients with advanced head and neck cancers [37, 38]. By using PET tracers which selectively concentrate in hypoxic cells (F-Miso) the visualization of hypoxic regions became possible [37, 39–44].

Retrospective data on head and neck cancer indicates that loco-regional control is already excellent with standard radio-chemotherapy if tumors without any sign of hypoxia on [18 F]-FMISO PET scans were treated. Lee et al. [45] showed for head and neck tumors with hypoxia detected by [18 F]-FMISO PET and treated using IMRT excellent loco-regional control (3y-LRPFS of 95%). Loco-regional control in head and neck tumors with significant enhancement in the [18 F]-FMISO PET was poor in a study by Thorwarth et al. [46]. Zips et al. showed a strong association for locally advanced head and neck cancer patients with stage III and IV of FMISO-image parameters and LPFS. The strongest association was seen for the PET investigations performed during week 1 and 2 of radiotherapy [47]. The stability of hypoxic tumor sub-volumes detected by FMISO-PET was investigated by Bittner et al. in 16 patients with LAHNSCC. They found in patients with hypoxic tumors in comparison between FMISO-PET 1 and 2 (two weeks apart) at the beginning of the radio-chemotherapy. In patients with persistent hypoxia after 2 weeks of treatment, the hypoxic sub-volumes showed relative geographical stable information [48].

According to this data, preferentially patients with significant tumor hypoxia would benefit from dose escalation. Since hypoxic sub-volumes are predominantly observed more centrally in the tumor, dose escalation in the central parts of the tumor, as done in the experimental arm of this study, should cover most hypoxic sub-volumes.

We hypothesize that patients with tumours exhibiting significant enhancement in the [18 F]-FMISO PET, will have a larger benefit from dose escalation than patients with no [18 F]-FMISO-enhancing tumours in terms of loco-regional tumor control.

IMRT provides a better sparing of normal tissues. In addition to better sparing of normal tissues, IMRT also allows escalating the radiation dose selectively to sub-volumes of the tumour. Simultaneously Integrated Boost technique (SIB-IMRT) is well described and evaluated especially regarding its acute and late toxicity [9, 30, 49–51]. This is true for salivary glands and for the mucosa [22–24, 27, 52–54].

Nevertheless, dose escalation to large parts of the tumor is inherently accompanied by the risk of more acute and late toxicity [28–31]. In consideration of this risk, dose escalation was restricted to 80.5 Gy in the current study, although theoretical considerations indicate that even higher doses could be required in hypoxic areas. A few other trials test dose escalation restricted to the [18 F]-FMISO PET positive regions of the tumours (“Dose Painting by Numbers”). In Denmark a phase I trial for FDG based dose painting up to 82 Gy in 2.34 Gy was initiated and first results were published [31].

## Abbreviations

AF: Accelerated Fractionation
AF + ccb: Accelerated Fractionation with Concomitant Boost
ARO: Arbeitsgemeinschaft Radioonkolgie
AUC: Area under the curve
BfArM: Bundesinstitut für Arzneimittel und Medizinprodukte (German Federal Institute for Drugs and Medical Devices)
BfS: Bundesamt für Strahlenschutz (German Federal Office of Radiation Protection)
CT: Computertomography
CTCAE: Common Toxicity Criteria Adverse Events
CTV: Clinical Target Volume
DFS: Disease Free Survival
DVH: Dose Volume Histogram
FDG: Fluorodesoxyglucose
FMISO: Fluoromisonidazol
GTV: Gross Target Volume
Gy: Gray
HF: Hyper-fractionation
IIT: Investigator initiated trial
IMRT: Intensity Modulated Radiotherapy
IMSE: Institut für Medizinische Statistik und Epidemiologie
*i*RT: Institut für Innovative Radiotherapie
LAHNSCC: Locally Advanced Head and Neck Squamous Cell Cancer
LRC: Loco-regional Control
MRI: Magnetic Resonance Imaging
MSZ: Münchner Studienzentrum
NF: Normo-fractionation
OAR: Organs at Risk
OS: Overall Survival
PET: Positron Emission Tomography
PFS: Progression Free Survival
PTV: Planning Target Volume
RT: Radiotherapy
RTOG: Radiation Therapy Oncology Group
SIB: Simultaneous Integrated Boost
VMAT: Volumetric Arc Therapy
